# Sevoflurane Suppresses Cardiomyocyte Pyroptosis in Myocardial Ischemia via NLRP3 Inflammasome Signaling

**DOI:** 10.1155/ancp/7119597

**Published:** 2025-08-24

**Authors:** Mingjing Feng, Lingling Zheng, Baozeng Chen, Huijian Shi

**Affiliations:** ^1^Department of Anesthesiology, The Second People's Hospital of Liaocheng, Liaocheng, Shandong, China; ^2^Department of Cardiology, Shengli Oilfield Central Hospital, Dongying, Shandong, China; ^3^Department of Cardiology, The Second People's Hospital of Liaocheng, Liaocheng, Shandong, China; ^4^Department of Anesthesiology, The Second Affiliated Hospital of Shandong First Medical University, Shandong, Jinan, China

**Keywords:** cardiomyocytes, myocardial ischemia, NLRP3 inflammatory vesicles, pyroptosis, sevoflurane

## Abstract

The purpose of this study was to investigate the impact of sevoflurane (SEV) on cardiomyocyte (CM) pyroptosis following myocardial ischemia (MI). Reverse validation was performed by pharmacologically activating NLRP3 with monosodium urate (MSU) to confirm that SEV's cardioprotective effects were specifically mediated through the NLRP3 inflammasome pathway. Sprague Dawley rats were randomly assigned to sham (sham), model (conventional anesthesia + MI-reperfusion [MIR] injury modeling), SEV (SEV inhalation anesthesia + MIR injury modeling), and SEV + NLRP3 (SEV inhalation anesthesia + MIR injury modeling + NLRP3) groups. The myocardial area at risk (MAAR) and the myocardial infarct size (MIS) were evaluated in each experimental group, and cardiac tissue was examined using hematoxylin-eosin (H&E), Masson trichrome, and TUNEL staining. The concentrations of creatine kinase-MB (CK-MB), cardiac troponin I (cTnI), oxidative stress (OS), and pyroptosis-associated proteins and various inflammatory markers in the serum and cardiac tissue were quantified. Results showed that compared to the sham group, both model and SEV groups exhibited a significant increase in MAAR and MIS, accompanied by severe histopathological damage and noticeable OS (*p*  < 0.05). Elevated levels of inflammatory factors, enhanced CM apoptosis, and increased expression of pyroptosis-associated proteins were also observed in these groups. Notably, the SEV intervention in the SEV group demonstrated evident mitigation of heart injury, reduced MAAR and MIS, diminished CM apoptosis and inflammatory factors, and suppressed pyroptosis-associated proteins. Additionally, we observed that NLRP3 activation significantly diminished the protective effects of SEV on MIR rats. This study uncovers a novel mechanism through which SEV suppresses CM pyroptosis by inhibiting NLRP3, as confirmed by pharmacological activation of NLRP3. This was evidenced by worsened histopathological damage, increased CM apoptosis, and higher levels of inflammatory factors, cardiac injury markers, and pyroptosis-associated proteins. Overall, SEV inhibits CM pyroptosis and mitigates OS and inflammation through the NLRP3 inflammasome.

## 1. Introduction

Myocardial ischemia (MI) is a pathological condition resulting from the narrowing of coronary arteries, leading to inadequate blood and oxygen supply to the myocardium [1, 2]. This condition primarily arises due to coronary artery stenosis. MI is especially prevalent during the perioperative period of cardiac surgery [3]. When coronary arteries become occluded, the myocardium suffers from oxygen deprivation, which, if prolonged, can result in myocardial cell death and necrosis [4, 5]. Recent clinical statistics reveal an alarmingly high incidence of perioperative cardiac MI, ranging from 8% to 37%, with an even higher prevalence among the elderly, affecting over 50% of this population [6]. Myocardial infarction, a consequence of MI, is a leading cause of mortality and the most common cause of chronic heart failure worldwide [7]. In the 1970s, Ginks et al. [8] conducted research involving MI-reperfusion mapping in dogs, discovering the effectiveness of reperfusion therapy in reducing myocardial injury following MI. Subsequently, reperfusion became widely regarded as the most effective treatment for MI. However, it is crucial to note that reperfusion can have adverse effects, exacerbating myocardial damage and potentially leading to cell death in patients with myocardial infarction [5, 9–12]. Therefore, it is advisable to consider the use of anesthetics with cardioprotective effects during cardiac surgery. This approach can help reduce the occurrence of MI and enhance the perioperative safety of patients [13].

Sevoflurane (SEV) is an inhalation drug used to induce and maintain general anesthesia in patients [14, 15]. It was demonstrated in several preclinical trials that using SEV postconditioning at the onset of reperfusion can mitigate ischemia/reperfusion injury, likely through the activation of the Akt/HIF-1α/VEGF signaling pathway [16–20]. SEV decreases biomarkers of heart injury through its anti-inflammatory actions by upregulating heat shock protein [21]. SEV lowers the incidence of reperfusion arrhythmias and enhances ventricular conduction [22]. Studies reveal that SEV contributes to better heart muscle function and reduces the infarcted area, thereby safeguarding the myocardium [23–25]. A recent study has proposed that SEV postconditioning protects myocardium in cardiac surgery under cardiopulmonary bypass, suggesting its potentially important role in MI [26]. Although all studies focused on cardioprotective effects of SEV, there are no studies detailing the effect of SEV on cell damage and underlying pathways that are involved during MIRI.

Pyroptosis is a type of planned cell death characterized by cell membrane rupture and the subsequent release of several inflammatory mediators [27, 28]. Clinically, in MI, the activation of the NLRP3 inflammasome, mediated by thioredoxin-interacting protein (TXNIP), plays a role in myocardial I/R injury [29, 30]. TXNIP induces NLRP3 inflammasome complex formation, mitochondrial stress-induced apoptosis, and pyroptosis [31].

Recent research suggested that SEV may impact acute lung damage by suppressing NLRP3-mediated pyroptosis [32]. Additionally, SEV provides cardioprotection by suppressing NF-κB activation in a heart perfusion model [33]. Moreover, Wu et al. [34] discovered that SEV can alleviate MI-reperfusion injury via the NLRP3 pathway. However, this research explored the role of SEV in inhibiting NLRP3-mediated pyroptosis in MIRI patients undergoing abdominal surgery. In this study, we focused on cardiomyocyte (CM) pyroptosis in a rat model via the NLRP3 inflammasome signaling pathway, assessing myocardial area at risk (MAAR), infarct size, inflammatory factors, and oxidative stress (OS) to explore the pathways involved in CM pyroptosis after MI. We hypothesize that SEV inhibits CM pyroptosis and counteracts OS through the NLRP3 inflammasome. Importantly, we further confirmed the specificity of SEV's effects by pharmacologically activating NLRP3, thereby providing direct evidence that the NLRP3 inflammasome is involved in SEV-mediated cardioprotection. Thus, our study contributes a nuanced perspective, enhancing our understanding of SEV's potential in addressing myocardial injury.

## 2. Materials and Methods

### 2.1. Animal Data

We acquired pathogen-free Sprague-Dawley rats, comprising both sexes and aged 10 weeks, with an average weight of 250 g ( ± 17 g), from Beijing Minhai Biotechnology Co., Ltd. (Animal License Number SYXK (Beijing) 2023-0033). The rats were housed in cages within a standard animal room, maintaining a constant temperature, and had unrestricted access to food and water. Our hospital's Animal Ethics Committee approved this study (Approval#33478, JGHA). The primary endpoint of the study was defined as major adverse cardiovascular events.

### 2.2. Establishment of MIRI Model

The MIRI rat model was created based on an early report by exposing and ligating the left anterior descending coronary artery [35]. The rats were sedated with an intraperitoneal injection of pentobarbital sodium at 40 mg/kg, which was followed by mechanical ventilation utilizing orotracheal intubation. The electrode was subcutaneously inserted into the limbs for continuous monitoring of heart conditions, while a ventilator was employed for respiratory support. An incision was made between the third and fourth ribs on the left chest wall, allowing for layered dissection. Ligation of artery was executed using sutures, followed by a 2 h reperfusion period. Reperfusion was initiated upon observation of an elevation of ST-area in the electrocardiogram. The successful establishment of MIRI model was confirmed by ST-segment resolution to 1/2 and observable reddening of the myocardium. Instances of death within 24 h after surgery were excluded from the scope of this experiment. At the end of the procedure, the surgical wounds of the rats were sequentially sutured. Rats subjected to these surgical procedures were classified into three groups: the model group (*n* = 20), SEV (*n* = 20), and SEV + NLRP3 (*n* = 10) group. The model group received standard anesthesia with pentobarbital sodium (50–70 mg/kg) injected intraperitoneally. To activate the NLRP3 inflammasome, the SEV + NLRP3 group received an intraperitoneal injection of monosodium urate (MSU, 10 mg/kg, Abcam, USA) following SEV treatment, The dose of MSU (10 mg/kg) was chosen based on previous studies that showed robust NLRP3 activation in rodent inflammation models [36]. Pilot experiments in our study confirmed that this dose effectively induced significant pyroptosis without causing systemic toxicity in rats; the SEV group received inhalation anesthesia with 3% SEV, and the SEV + NLRP3 group received inhalation anesthesia with 3% SEV followed by an intraperitoneal injection of 10 mg/kg MSU (Abcam, USA). The sham operation group underwent a suture operation without coronary artery ligation, following the same procedural steps as the model and SEV groups. Subsequently, the animals were reintroduced to their cages after fully recovering from anesthesia. Two hours after the intervention, each group's heart rate (HR), mean arterial pressure (MAP), and rate-pressure product (RPP) values were measured using a noninvasive sphygmomanometer (BP2006A model).

### 2.3. Measurement of MAAR and Myocardial Infarct Size (MIS)

The left anterior descending coronary artery was blocked to maintain ischemic conditions. The heart was then collected and installed on the Langendorff isolated heart perfusion apparatus. After continuous saline irrigation for 1 min, the left ventricle was isolated and refrigerated at −20°C for 15 min. Subsequently, the left ventricle was sliced into 2 mm cross-cut sections, immersed in a phosphate buffer solution (PBS) water bath, and examined. The ischemic infarct area (pale), ischemic but noninfarcted area (red), and the normal area were distinguished. The percentage area of left ventricular area at risk (left ventricular area at risk = noninfarcted area + infarct area) and the infarct size (infarct size = size of ischemic infarct area/size of left ventricular area at risk) were then calculated. The rats were euthanized using 3% isoflurane followed by cervical dislocation. All experimental procedures were conducted in accordance with the ARRIVE guidelines.

### 2.4. Hematoxylin-Eosin (H&E) of Cardiac Tissue Staining

Cardiac tissues were harvested from each rat, preserved in 4% paraformaldehyde (Sigma# 1004960700), and then embedded in paraffin. The cardiac tissue blocks obtained were sectioned into 4 μm slices, which were then placed on charged slides. H&E staining was performed on these sections. Briefly, the slides underwent deparaffinization using xylene, followed by rehydration through different alcohol concentrations and water. Nuclei were stained with hematoxylin (Millipore Sigma#517-28-2), excess solution was rinsed off, and the cytoplasm was stained with eosin (Sigma# HT110232). The slides were dehydrated through increasing alcohol concentrations, dehydrated in xylene changes, and dried before sealing with mounting media (Abcam#ab104141) and cover slips for microscopic observation (Axiovert 200M, Zeiss).

### 2.5. Masson Trichrome Staining

Cardia tissue sections underwent deparaffinization and rehydration in series of alcohol and finally passed through Weigert's iron hematoxylin (Sigma# HT1079-1SET) for 3 minutes before washing. Sections were then stained for 10–15 min with Biebrich scarlet-acid fuchsin (Sigma # HT151-250 ML). Sections were immediately transferred to aniline blue (Sigma#B8563) for 5–10 min and briefly washed and differentiated in 1% acetic acid (Sigma#64197) for 2–5 min. Final steps involved rapid dehydration, clearing in xylene, and mounting of slides with mounting medium.

### 2.6. TUNEL Assay

The apoptosis detection kit (Takara, cat. #MK500) was utilized to perform TUNEL staining. Briefly, the heart tissue sections underwent xylene deparaffinization, were rehydrated in progressively lower alcohol concentrations, and were digested for 10 min using proteinase K. The slides were treated with the TUNEL reaction mixture for an hour at 37°C in a humidified chamber to detect positive cells, and then they were treated with DAPI for a few minutes to detect nuclei. Apoptotic cells were counted in five randomly selected fields per section at 10× magnification, with 100 cells counted in each field. Cells were considered positive if they showed nuclear DAPI/TUNEL co-staining.

### 2.7. Content of Myocardial Injury Markers

Following anesthesia induction, 3 mL of cardiac blood was drawn from each group of rats through the inferior vena cava. Serum was obtained by centrifugation and then it was analyzed for creatine kinase-MB (CK-MB) and cardiac troponin I (cTnI) using an automatic biochemical analyzer (Coulter JTIR, USA).

### 2.8. Serum Inflammatory Factors and OS

An ELISA kit (Shanghai Xuanya Biotechnology Co., Ltd) was used to measure levels of NF-ɡB, TNF-α, IL-6, SOD, and MDA. The testing procedure strictly adhered to the kit instructions. Briefly, the diluted gradient standard was introduced to the ELISA-coated plate, followed by the addition of tissue lysate, and subsequent incubation with the enzyme labeling reagent. After washing, color development was initiated by adding the color developer buffer, and the reaction was allowed to proceed in the absence of light at 37°C for 15 min. The stop buffer was then added, and each well's optical density was measured at 450 nm using a microplate reader within 15 min. The contents of markers in the rats' cardiac tissue were finally detected based on the OD values obtained.

### 2.9. Detection of Pyroptosis-Associated Proteins

Total protein of heart tissue was extracted using the bicinchoninic acid (BCA) method and underwent denaturation and electrophoresis on SDS-PAGE. The proteins were then transferred onto a PVDF membrane, followed by sealing with 5% skimmed milk powder and overnight incubation with primary antibodies at 4°C. The membranes were washed three times with TBS the following day, and respective secondary antibodies (antirabbit, ThermoFisher, #31460, 1:5000, and antirat, ThermoFisher#31470, 1:5000) were added for 2 h. Membrane development was achieved through enhanced chemiluminescence (ECL), and images were captured. The quantification of resultant bands was accomplished using ImageJ software. Primary antibodies were procured from Abcam and cell signaling technology, with specific antibodies targeting NLRP3 (rabbit, ab#263899, 1:800), IL-1β (rabbit, ab#9722, 1:1000), IL-18 (rabbit, ab#191152, 1:700), GSDMD-N/FL (rabbit, ab#155233, 1:500), caspase-1 (rabbit, ab#155233, 1:500), caspase 4 (rabbit, CST#4450, 1:200), caspase 5 (rabbit, CST#4429, 1:400), and caspase 11 (Rat, CST#14340, 1:900), and the housekeeping protein β-actin (rabbit, ab#6276, 1:5000). Original western blots associated with this study can be found in Supporting Information S1.

### 2.10. Statistical Analysis

The Data was analyzed using SPSS 24.0 software and presented as the mean ± SD. The one-way ANOVA followed by a Bonferroni post hoc test was used for multiple-group comparisons. A *p*-value of < 0.05 was considered statistically significant.

## 3. Results

### 3.1. Comparison of Hemodynamics, MAAR, and MIS

**Figure 1:**
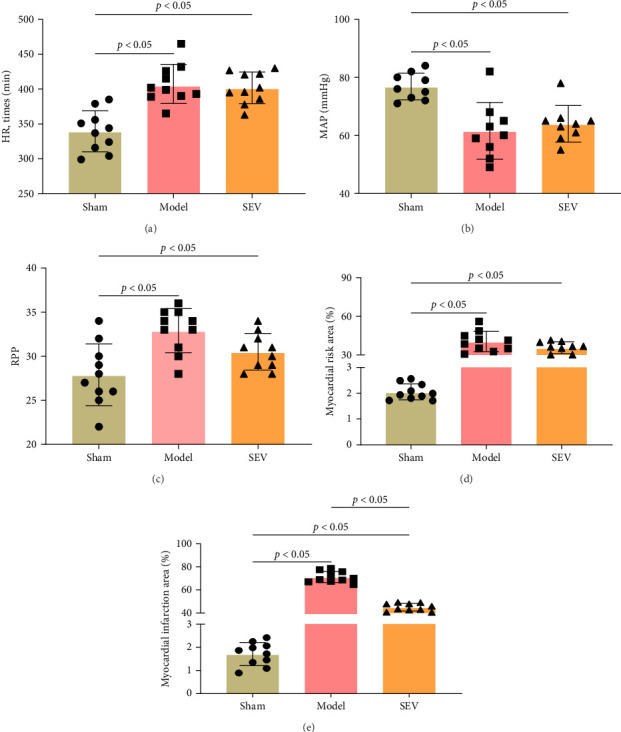
Comparative analysis of hemodynamic parameters and myocardial injury (*n* = 10). HR, MAP, RPP, MAAR, and MIS were evaluated between sham, model, and SEV group. (A) Comparison of HR. (B) Comparison of maps. (C) Comparison of BRP. (D) Comparison of myocardial risk area. (E) Comparison of myocardial infarction area. Data are represented as mean ± SD and analyzed using one-way ANOVA followed by Bonferroni post hoc test. *p*=0.34 represents a statistical difference between sham vs. model and sham vs. SEV groups.

Following the categorization into sham, model, and SEV intervention groups, we conducted a thorough evaluation of hemodynamic parameters in these groups, including HR, MAP, and RPP. Additionally, we assessed the extent of MAAR and MIS in each group of rats. The findings revealed no discernible variations in HR, MAP, and RPP between the model and SEV groups (Figure 1). While both the model and SEV groups showed higher HR and RPP, the sham group demonstrated higher MAP than both the model and SEV groups. Nonetheless, all these parameters exhibited statistically significant differences when compared to the sham group. Furthermore, we detected statistically lower MAAR and MIS in the sham group than the other two groups. The MAAR did not show a statistically significant difference between the model and SEV groups; however, the MIS was lower in the SEV group than in the model group. SEV intervention does not significantly alter hemodynamic parameters, but contributes to a reduced myocardial infarct size, suggesting a potential cardioprotective effect in the context of ischemia.

### 3.2. Comparison of Pathological Changes of Cardiac Tissue

**Figure 2:**
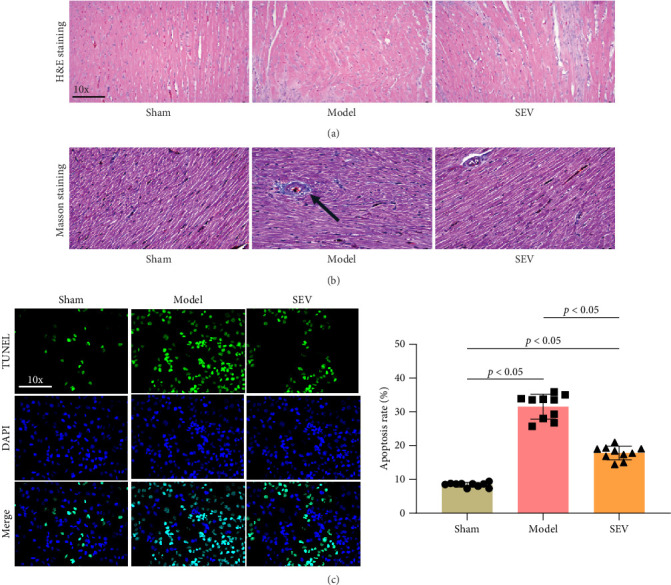
Histological assessment of cardiac tissues (*n* = 10). (A) Representative images of H&E staining depicting myocardial morphology in the sham, model, and SEV groups. (B) Masson staining illustrating fiber deposition in cardiac tissues of each experimental group. (C) TUNEL staining indicating the apoptotic rate of myocardial cells in heart tissues for each group. Data are represented as mean ± SD and analyzed using one-way ANOVA followed by Bonferroni post hoc test. *p*  < 0.01 represents a statistical difference between sham vs. model, sham vs. SEV group, and model vs. SEV group.

The cardiac tissues from each experimental group were dissected and subjected to H&E and Masson staining to assess the morphological characteristics and fiber deposition of myocardial cells. The findings revealed that the sham group exhibited well-defined boundaries, regular morphology, organized muscle fibers, and an absence of cell edema, necrosis, infarction lesions, or significant fiber deposition (Figure 2A,B). Conversely, the model group displayed myocardial necrosis, characterized by swollen or necrotic myocardial cells, disordered alignment, and ruptured myocardial fibers. In comparison, the SEV group demonstrated a notable improvement in cellular pathology compared to the model group (Figure 2A,B). The evaluation of myocardial cell apoptosis, assessed through TUNEL staining (Figure 2C), indicated a significantly higher apoptotic rate in the model group compared to the other groups. Furthermore, the SEV group exhibited a marked suppression in myocardial apoptosis compared to the model group (Figure 2C), suggesting a considerable inhibitory effect on cardiac apoptosis in MIRI rats.

### 3.3. Detection of Myocardial Injury Markers, Inflammatory Factors, and Myocardial Enzymes

**Figure 3:**
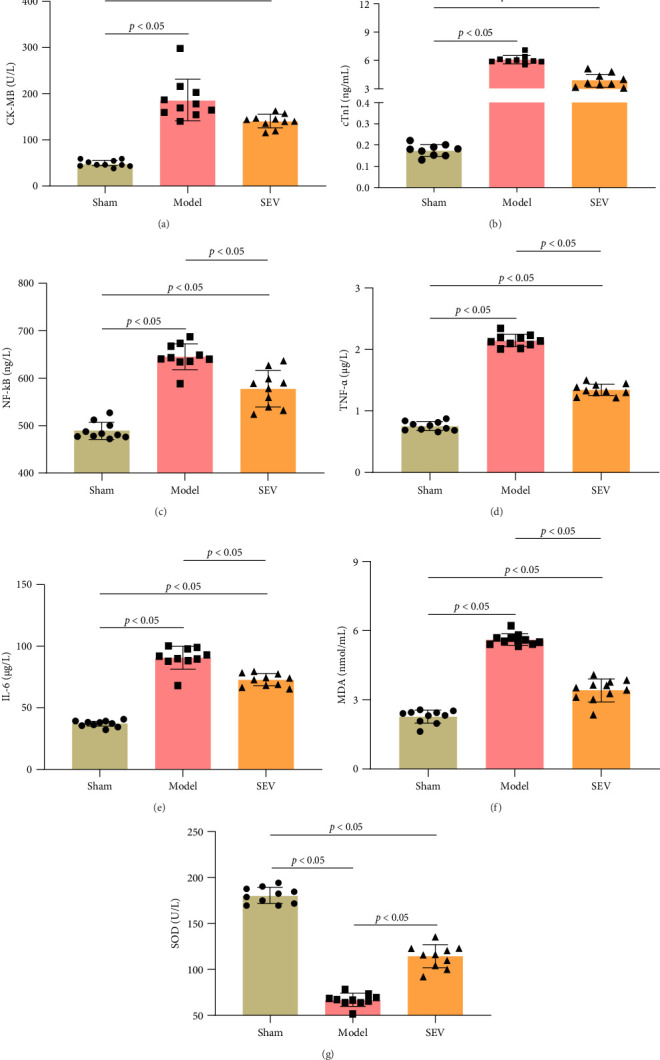
Evaluation of myocardial biomarkers release post myocardial MIRI (*n* = 10). (A, B) CK-MB and cardiac troponin levels in serum compared among sham, model, and SEV groups. The quantitative assessment showing a significant increase in myocardial enzyme release in groups, which was reversed by SEV intervention (*p*  < 0.01). (C–G) ELISA was used to evaluate cardiac biomarkers, for example, NF-κB, TNF-α, IL-6, MDA, and SOD. Sham group had lowest NF-κB, TNF-α, IL-6, MDA, and highest SOD. SEV group exhibited reduced NF-κB, TNF-α, IL-6, MDA, and increased SOD vs. model. Data are represented as mean ± SD and analyzed using one-way ANOVA followed by Bonferroni post hoc test. *p*  < 0.05 represents a statistical difference between sham vs. model, sham vs. SEV group, and model vs. SEV group.

Following MI, myocardial cells are damaged, resulting in the release of intracellular enzymes as a result of membrane rupture. The enzyme content in serum or medium can be used to determine the severity of cardiac cell damage. CK-MB is the main enzyme released into the bloodstream following CM damage, and concurrently, cTnI is also released extracellularly. Serum levels of CK-MB and cTnI act as direct indications of the severity of cardiac function injury in rats. The model group released much more cardiac enzymes than the Sham group, indicating severe cardiac damage (Figure 3A,B). Additionally, the SEV group had a lower release of cardiac enzymes than the model group, suggesting that SEV therapy may reduce the release of myocardial enzymes following MIRI. The quantity of NF-κB, TNF-α, IL-6, MDA, and SOD in cardiac tissues was measured by ELISA (Figure 3C–G). The sham group had considerably reduced levels of NF-κB, TNF-α, IL-6, and MDA compared to the model and SEV groups (Figure 3C–F), with SOD being the highest (Figure 3G, *p*  < 0.05). Notably, the SEV group exhibited reduced levels of these markers with an increase in SOD levels compared to the model group, suggesting the potential positive impact of SEV intervention in myocardial injury post MIRI.

### 3.4. Comparison of CMs Pyroptosis

**Figure 4:**
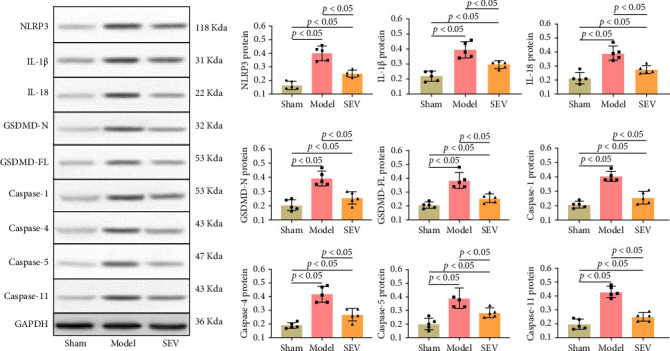
Impact of sevoflurane on cardiomyocyte pyroptosis (*n* = 5). The levels of pyroptosis-related proteins were assessed in sham, model, and SEV groups to investigate the influence of SEV on cardiomyocyte pyroptosis. Data are represented as mean ± SD and analyzed using one-way ANOVA followed by Bonferroni post hoc test. *p* < 0.05 represents a statistical difference between sham vs. model, sham vs. SEV group, and model vs. SEV group.

Pyroptosis has been linked to cell death and the regulation of MIRI [37]. We thus investigated the impact of SEV on CM pyroptosis by accessing the levels of pyroptosis-related proteins in the cardiac tissue. The analysis revealed that the sham group exhibited the lowest levels of pyroptosis-related proteins (Figure 4), including NLRP3, IL-1β, IL-18, GSDMD-N, GSDMD-FL, and caspases (Caspase-1, Caspase-4, Caspase-5, and Caspase-11). In contrast, the model group, which represents the MIRI condition, showed significantly elevated levels of these proteins, indicating heightened pyroptosis activity (Figure 4). Interestingly, the introduction of SEV in the treatment group resulted in a marked reduction in the levels of these pyroptotic markers compared to the model group. Specifically, SEV treatment significantly lowered the expression of NLRP3, IL-1β, IL-18, GSDMD-N, GSDMD-FL, and caspases (Figure 4). In conclusion, SEV demonstrates significant protective effects against CM pyroptosis by reducing the levels of pyroptosis-related proteins in the cardiac tissue.

### 3.5. Evaluation of CMs Pyroptosis After NLRP3 Activation

**Figure 5:**
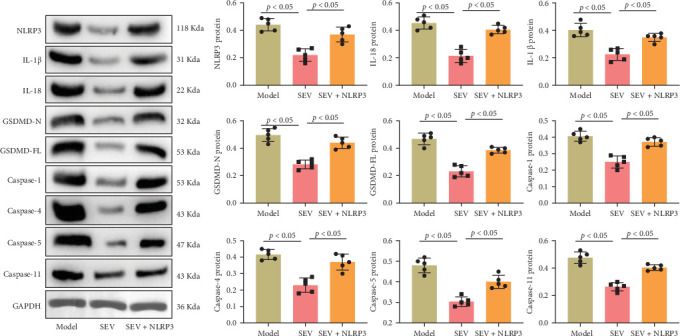
Effects of NLRP3 activation on cardiomyocyte pyroptosis (*n* = 5). The levels of pyroptosis-related proteins in model, SEV, and SEV + NLRP3 groups were assessed using western blot. Data are represented as mean ± SD and analyzed using one-way ANOVA followed by Bonferroni post hoc test. *p* < 0.05 represents a statistical difference between model vs. SEV and SEV vs. SEV + NLRP3 groups.

We evaluated whether SEV protects the rat heart from I/R injury by inhibiting NLRP3-mediated pyroptosis. Western blot analyses revealed significant insights into the effects of SEV and NLRP3 activation on pyroptosis-related proteins in MIRI rats (Figure 5). The results demonstrated that SEV treatment significantly reduced the cardiac levels of key pyroptosis markers, including NLRP3, IL-1β, IL-18, GSDMD-N, GSDMD-FL, and caspases (Caspase-1, Caspase-4, Caspase-5, and Caspase-11). Specifically, SEV-treated groups showed a marked decrease in NLRP3, IL-1β, and IL-18 proteins compared to the model group, indicating an anti-inflammatory effect of SEV (Figure 5). Similarly, the levels of GSDMD-N and GSDMD-FL, which are indicative of pyroptotic cell death, were significantly lowered in the SEV-treated group. Additionally, SEV effectively reduced the expression of caspases involved in the pyroptosis pathway (Figure 5). However, the protective effects of SEV were reversed upon activation of NLRP3 with MSU. The SEV + NLRP3 group exhibited increased levels of NLRP3, IL-1β, IL-18, GSDMD-N, GSDMD-FL, and caspases compared to the SEV group, suggesting that NLRP3 activation reinstates the pyroptotic pathway (Figure 5). This reversal underscores the pivotal role of NLRP3 in mediating pyroptosis and highlights that SEV's cardioprotective effects are closely linked to its ability to inhibit NLRP3 activation. In conclusion, SEV exhibits a significant cardioprotective effect by inhibiting NLRP3-mediated pyroptosis in MIRI rats. The reversal of this effect by NLRP3 activation confirms the critical role of the NLRP3 inflammasome in cardiac pyroptosis.

### 3.6. NLRP3 Activation Negated the Defensive Effects of SEV on Cardiac Myocardial Injury

**Figure 6:**
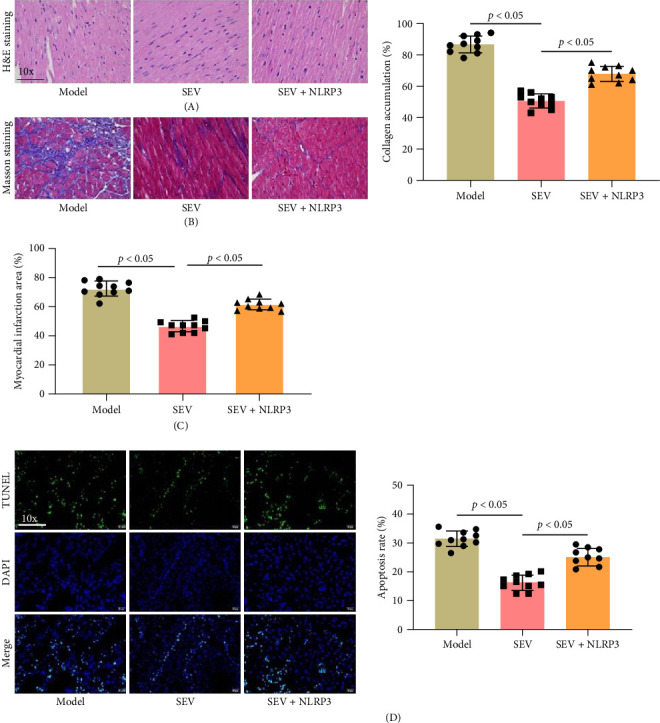
Histological assessment of cardiac tissues after NLRP3 activation (*n* = 10). (A) H&E staining showed myocardial morphology in the model, SEV, and SEV + NLRP3 groups. (B) Masson staining showed fiber deposition in cardiac tissues of rats. (C) The size of myocardial infarct was evaluated in rats of different groups. (D) TUNEL staining was used to evaluate the apoptotic rate of myocardial cells in rat heart tissues in each group. Data are represented as mean ± SD and analyzed using one-way ANOVA followed by Bonferroni post hoc test. *p* < 0.05 represents a statistical difference between model vs. SEV and SEV vs. SEV + NLRP3 groups.

We further examined the impact of NLRP3 activation on MIRI rats treated with SEV. H&E and Masson staining demonstrated that SEV treatment alleviated disordered myocardial fibers and inflammatory cell infiltration, but these benefits were reversed by NLRP3 activation (Figure 6A,B). Additionally, the size of myocardial infarction, which was reduced in the SEV group, increased with NLRP3 activation compared to the SEV group (Figure 6C). Moreover, TUNEL staining revealed higher apoptotic rate in the SEV + NLRP3 group compared to the SEV group (Figure 6D). These findings indicate that NLRP3 activation diminishes the protective effects of SEV against myocardial infarction and apoptosis.

### 3.7. NLRP3 Activation Reverses SEV's Protective and Anti-Inflammatory Effects in MIRI Rats

**Figure 7:**
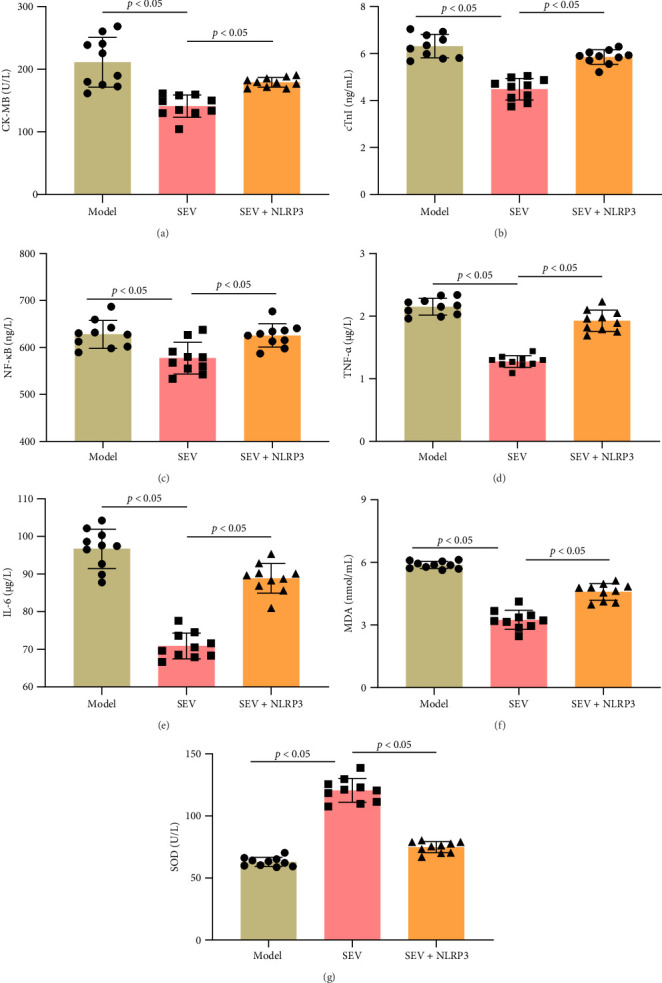
Effects of NLRP3 activation on inflammatory response and myocardial biomarkers release postmyocardial MIRI (*n* = 10). (A, B) CK-MB and cTnI levels in rat serum in each group. (C) NF-κB, (D) TNF-α, (E) IL-6, (F) MDA, and (G) SOD levels were assessed by ELISA. Data are represented as mean ± SD and analyzed using one-way ANOVA followed by Bonferroni post hoc test. *p* < 0.05 represents a statistical difference between model vs. SEV and SEV vs. SEV + NLRP3 groups.

We next examined the expression of myocardial cell injury indicators, especially CK-MB and cTnI. Our findings showed that blood levels of CK-MB and cTnI were considerably lower in the SEV group than in the model group. However, these markers were higher in the SEV + NLRP3 group compared to SEV group (Figure 7A,B). Moreover, we used ELISA to detect the levels of NF-κB, TNF-α, IL-6, MDA, and SOD in rat cardiac tissues. SEV group had significantly lower levels of NF-κB, TNF-α, IL-6, and MDA compared to the model group, indicating reduced inflammation. However, these inflammatory markers were significantly elevated in the SEV + NLRP3 group compared to the SEV group (Figure 7C–F). Conversely, the SOD levels, which indicate antioxidant activity, were higher in the SEV group and significantly reduced in the SEV + NLRP3 group (Figure 7G). These findings imply that SEV protects against myocardial infarction after MIRI by inhibiting the NLRP3 signaling pathway, and that NLRP3 activation can reverse these protective anti-inflammatory effects.

## 4. Discussion

This study explored the impact of SEV on MIRI and elucidated the underlying mechanisms for inhibiting MIRI in vivo. Our study demonstrated that SEV exerted protective effects by moderating CM inflammation and pyroptosis, primarily via the NLRP3 signaling pathway. The identification of this potential mechanism underscores the clinical significance of SEV, suggesting its promising role in both preventing and reversing MIRI.

Previous research has extensively investigated the mechanisms underlying MIRI, with a significant focus on understanding the impact of inflammatory factors in ischemia-reperfusion injury [38]. Proinflammatory factors are upregulated during MI-reperfusion [39], triggering a cytokine cascade reaction involving TNFα, IL-6, and IL-1β [40, 41]. Furthermore, MIRI is related to enhanced reactive oxygen species generation, calcium overload in CMs, induction of inflammatory mediators and chemokines upon reperfusion, ultimately leading to apoptosis [42]. Clinical emphasis is placed on attenuating inflammatory responses and reducing OS to offer myocardial protection [43].

In clinical applications, SEV has demonstrated a protective effect against MI injury . Employed in general anesthesia, SEV exhibits rapid onset and patient recovery, minimal influence on hemodynamics, and few toxic side effects. Yu et al. [44] suggest that SEV, at varying concentrations, may impact memory and cognitive function, hinting at potential effects on MIRI, encompassing myocardial preservation and neuroprotective effects. However, the precise mechanism through which SEV acts against MI remains undetermined.

To ascertain SEV's protective impact on the myocardium and its underlying mechanisms, we established sham, model, and SEV groups. Evaluation of serum injury factors in rats revealed that SEV's effect on MI injury is associated with anti-inflammatory properties and the inhibition of pyroptosis. Hemodynamic tests revealed no significant variations in HR, MAP, or RPP between the model and SEV groups, suggesting comparable anesthetic effects without inducing noticeable stress changes in rats. Further examination of cardiac tissue damage indicated significantly higher MAAR and MIS in the model group compared to the sham group. While SEV intervention did not affect MAAR, it significantly reduced MIS compared to the model group. Additionally, SEV group demonstrated substantial reductions in histopathological damage and myocardial apoptosis compared to the model group.

In our study, elevated levels of interleukins, NF-κB, TNF-α, caspases, MDA, and heightened myocardial injury markers (CK-MB and cTnI), along with reduced SOD, were observed in the model group. Notably, an increased level of NLRP3 and GSDMD-N/FL in the model group confirmed the presence of significant CM pyroptosis. However, in the SEV group, heart injury was markedly ameliorated, with reductions in CM pyroptosis and expression of NLRP3, NF-κB, TNF, IL-1β, IL-18, GSDMD-N/FL, MDA, and Caspase-1/4/5/11 proteins. This inhibition results in the reduction of the inflammatory response and cell pyroptosis, ultimately improving CM function. These findings underscore the exceptional myocardial protective effects of SEV, effectively reversing the deterioration of cardiac function induced by MIRI, inhibiting inflammatory reactions and OS, and blocking CM pyroptosis.

Previous research has highlighted a significant upregulation of NLRP3, cleaved caspases, IL-1β, IL-18, and GSDMD N-terminal fragments in CMs [45]. GSDMD plays a pivotal role in cell pyroptosis, where Caspase-1 cleaves GSDMD (GSDMD-FL) to generate the GSDMD-N-terminal, forming a 10–14 nm pore in the cell membrane. Subsequently, Caspase-1 cleaves Pro-IL-1β and Pro-IL-18 and secretes IL-1β and IL-18, resulting in inflammatory responses, ultimately leading to cell death [46, 47]. The inflammasome activation and subsequent signaling of inflammatory molecules form a pivotal pathway that triggers myocardial inflammation after ischemia [48]. The interplay between pyroptosis occurrence and the release of IL-1β and IL-18 exacerbates MIRI. Unlike alternative cell death processes, pyroptosis stands out as a defensive mechanism triggered by pathogenic infections [49].

Consistent with this perspective, Shu and Du [50] proposed in their research that SEV mitigated brain injury by inhibiting the pyroptosis of neurons. A previous study has also indicated that SEV can activate Akt through PI3K activation, leading to downstream phosphorylation of various factors. This process reduces mitochondrial permeability transition pore opening, regulates the expression of proapoptotic and antiapoptotic proteins, thus inhibiting cell apoptosis and diminishing the myocardial infarction area [51]. In our study, we demonstrated that the activation of NLRP3 signaling mitigated the cardioprotective effects of SEV against histopathological damage, CM survival and pyroptosis, and inflammatory response in rat cardiac injury in vivo, suggesting that SEV attenuated the MI-reperfusion injury via the NLRP3-mediated pyroptosis.

While our study offers valuable insights into the cardioprotective effects of SEV against MI, it is important to recognize some limitations that may affect the generalizability of our findings. Firstly, our investigation primarily relies on a rat model, and translating these results to clinical settings requires careful consideration. Additionally, the focus on the NLRP3 inflammasome pathway, while providing a specific mechanism, may not encompass the entirety of SEV's cardioprotective actions. Furthermore, based on our current data, we cannot determine whether SEV downregulates NLRP3 at the transcriptional or translational level, which remains an important avenue for future investigation.

Future research endeavors should aim to address these limitations by diversifying experimental models, potentially incorporating larger animal models or human studies. Exploring additional molecular pathways associated with SEV-induced cardio-protection could provide a more comprehensive understanding of its mechanisms. Cellular assays should be conducted to elucidate how SEV modulates NLRP3 expression and whether it directly influences NLRP3 inflammasome assembly. Moreover, investigating the long-term effects and safety profile of SEV in clinical scenarios would be pivotal for establishing its therapeutic potential in mitigating MI-reperfusion injury.

In summary, our findings elucidate that SEV's safeguarding effect against MIRI involves anti-inflammatory and anti-OS mechanisms by restraining CMs' pyroptosis.

## Data Availability

The datasets are available from the corresponding author upon request.

## Supporting Information

Supporting Information Original western blots associated with this study can be found as Supporting Information. File S1. This file contains the original full-length western blot images and ladder corresponding to the cropped figures presented in the manuscript.
