# Supplementary figures and images for: Sevoflurane Suppresses Cardiomyocyte Pyroptosis in Myocardial Ischemia via NLRP3 Inflammasome Signaling
Source: Anal Cell Pathol (Amst). 2025 Aug 24;2025:7119597. doi: 10.1155/ancp/7119597 (PMC12399354; doi:10.1155/ancp/7119597)

**Actin**


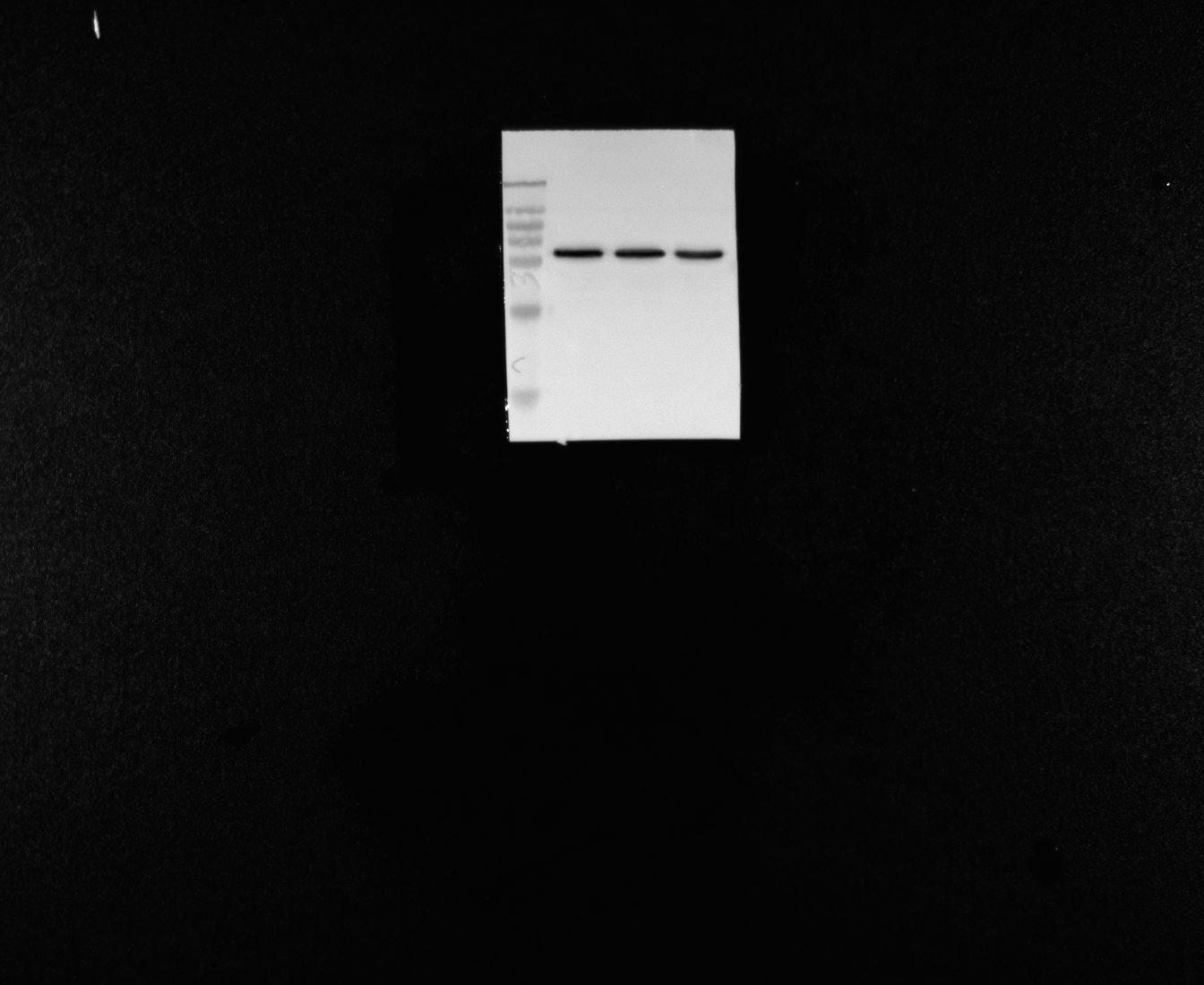


**Caspase 1**


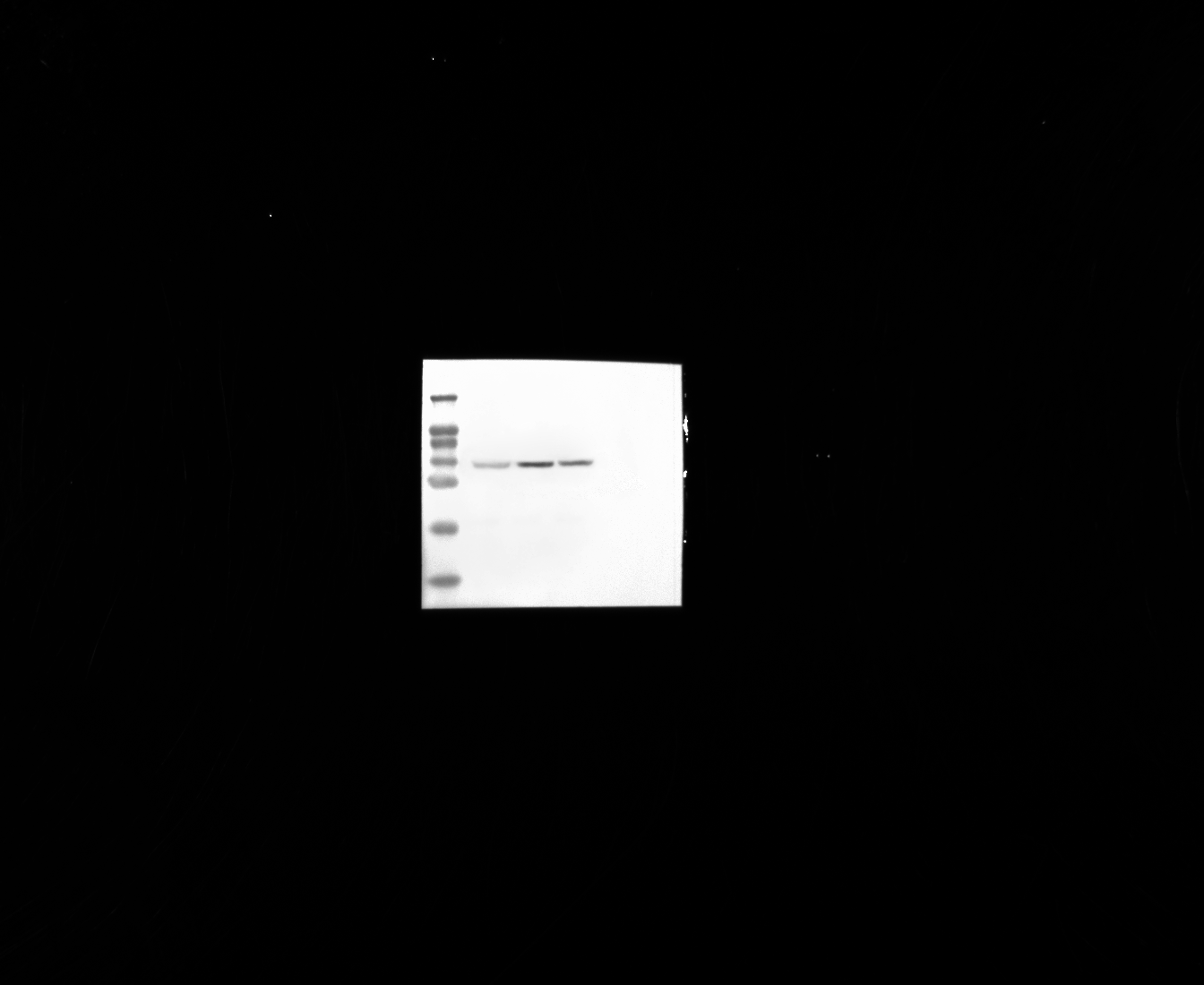


**Caspase 4**


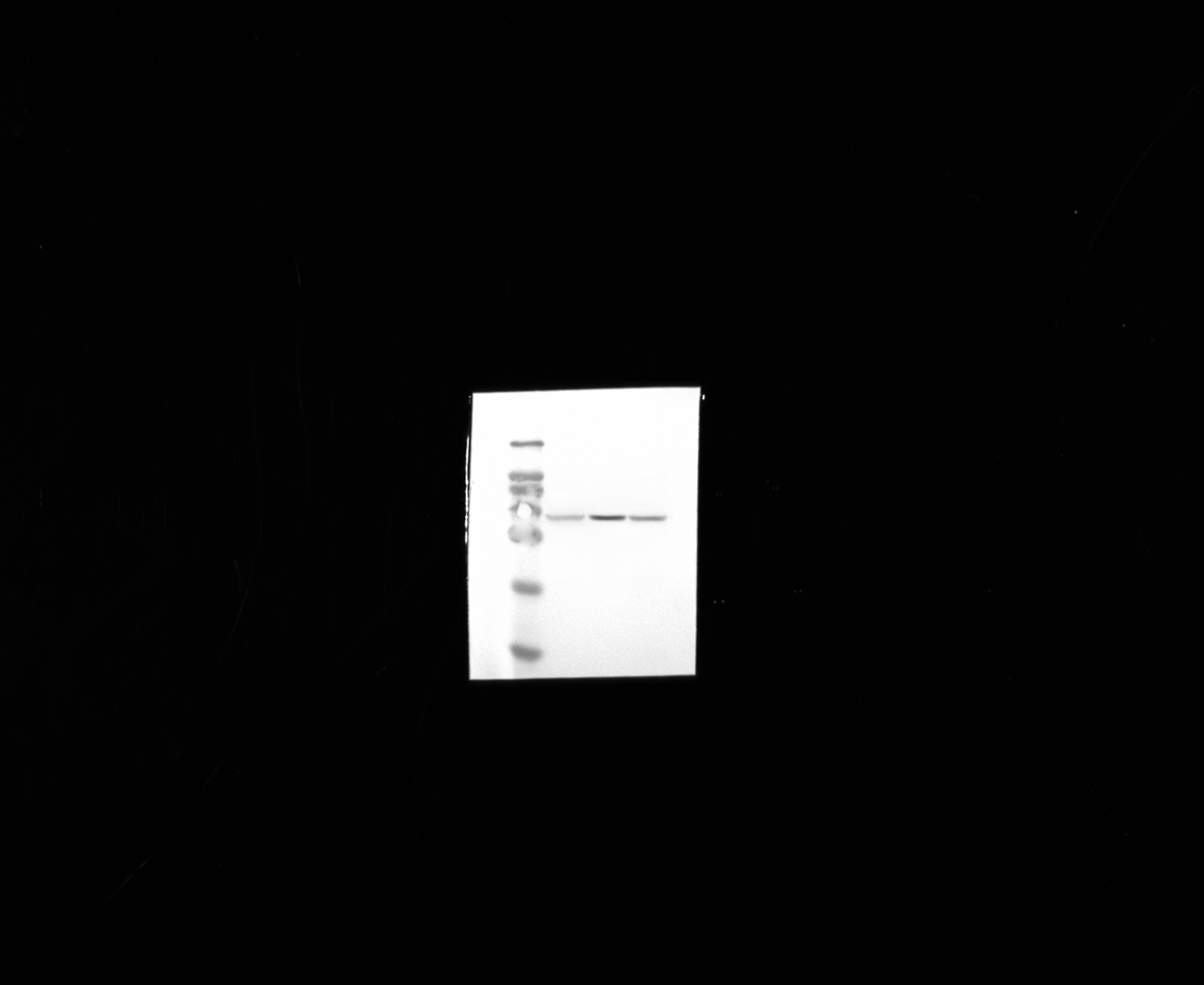


**Caspase 5**


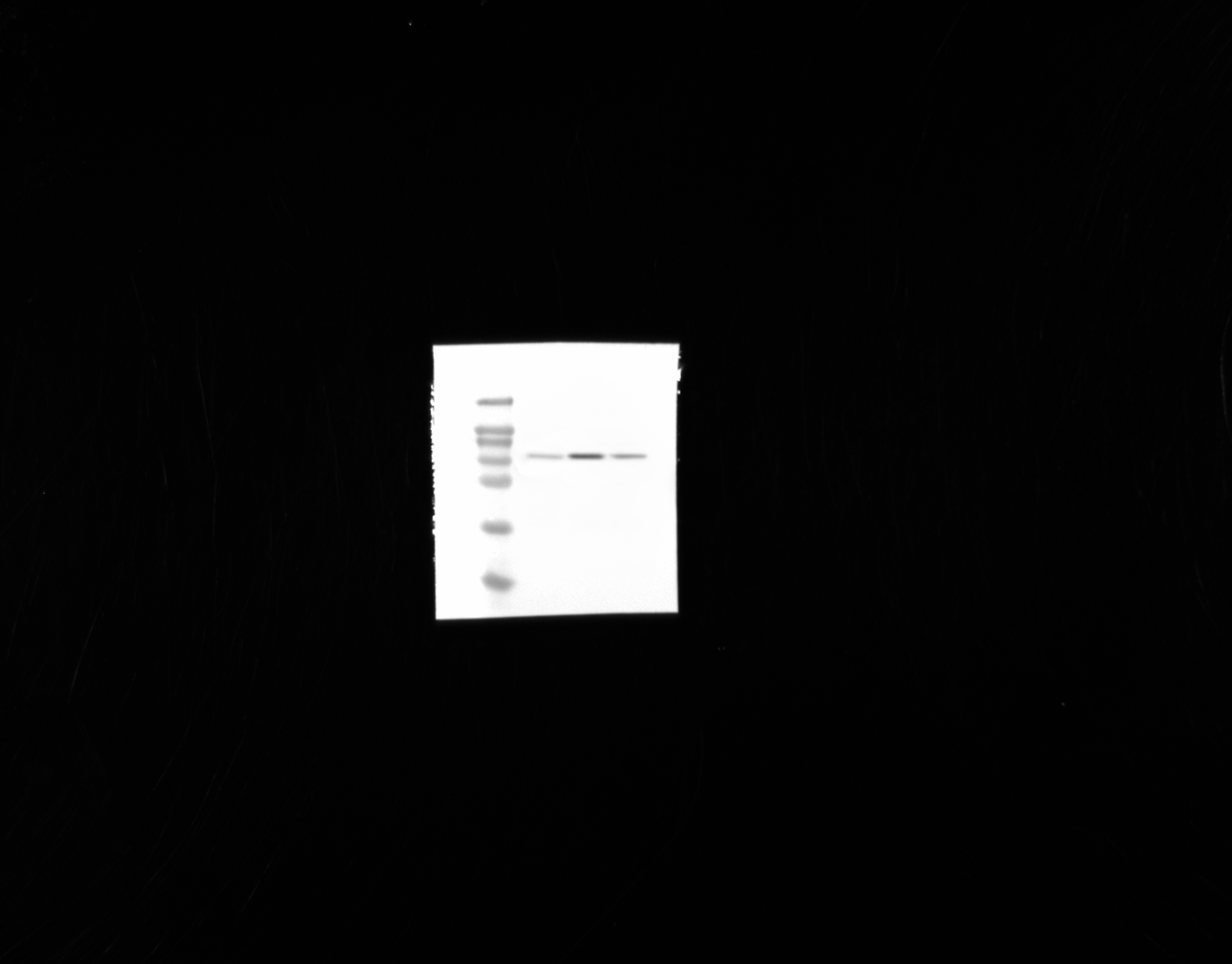


**Caspase 11**


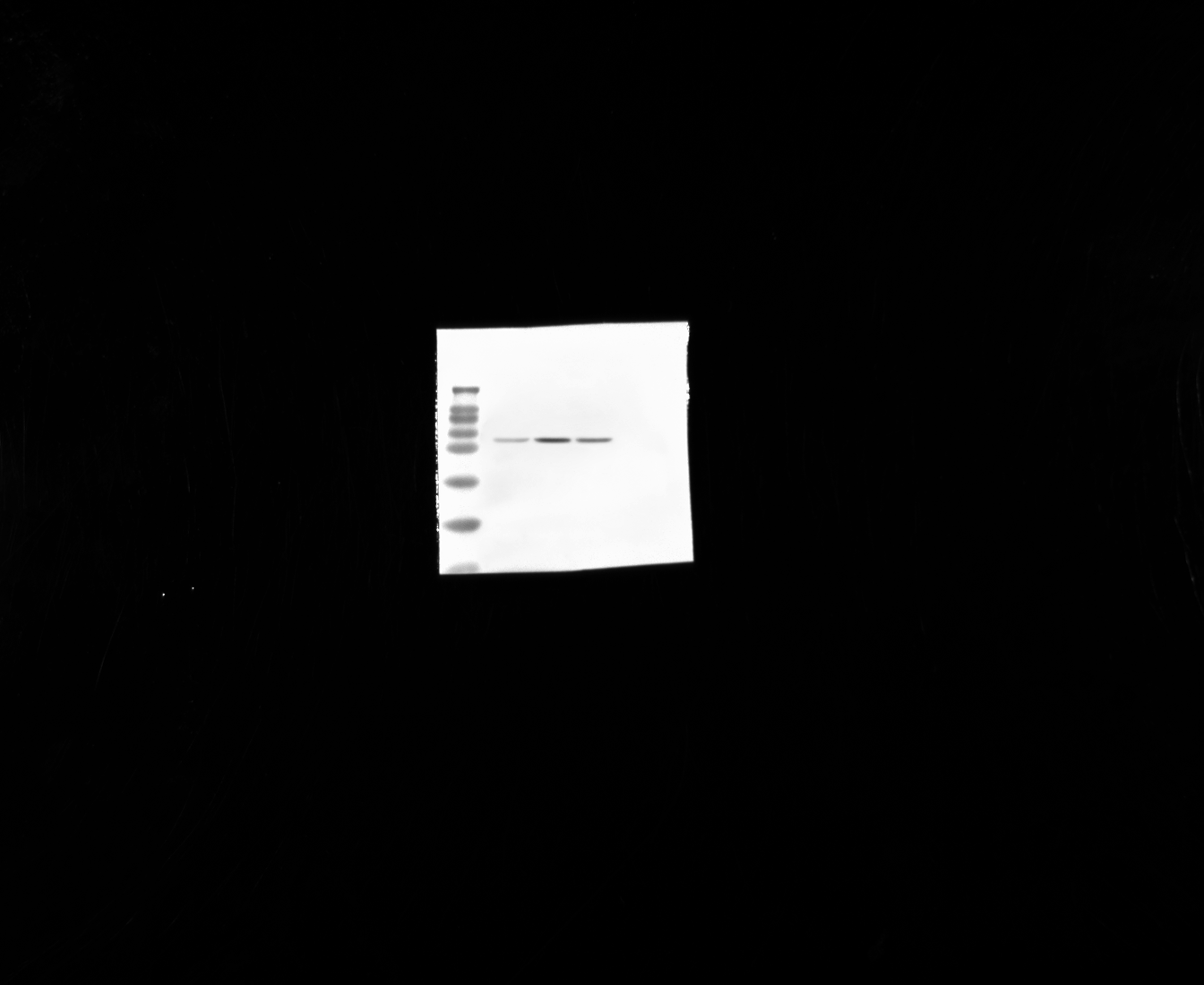


**GSDMD-FL**


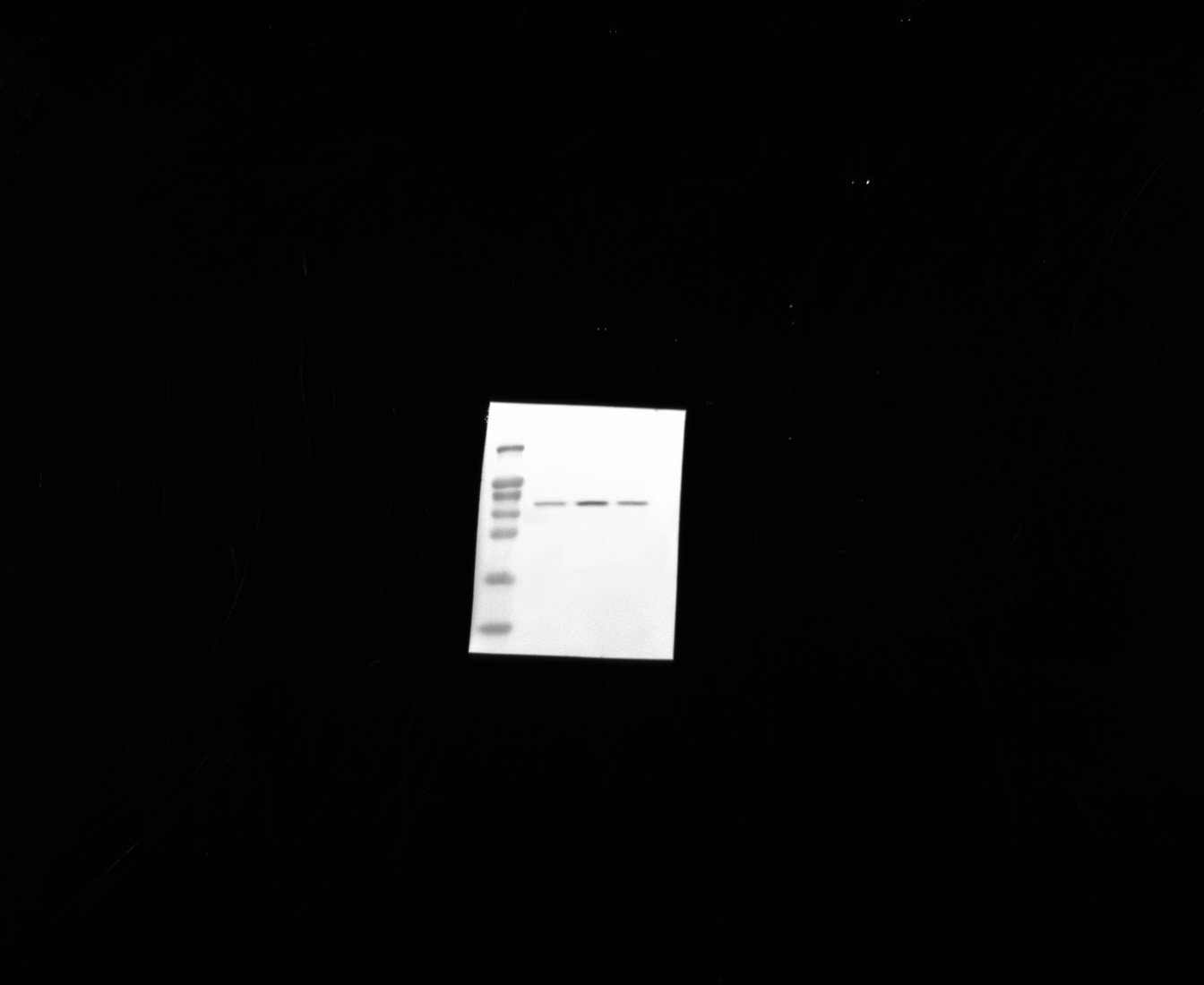


**GSDMD-N**


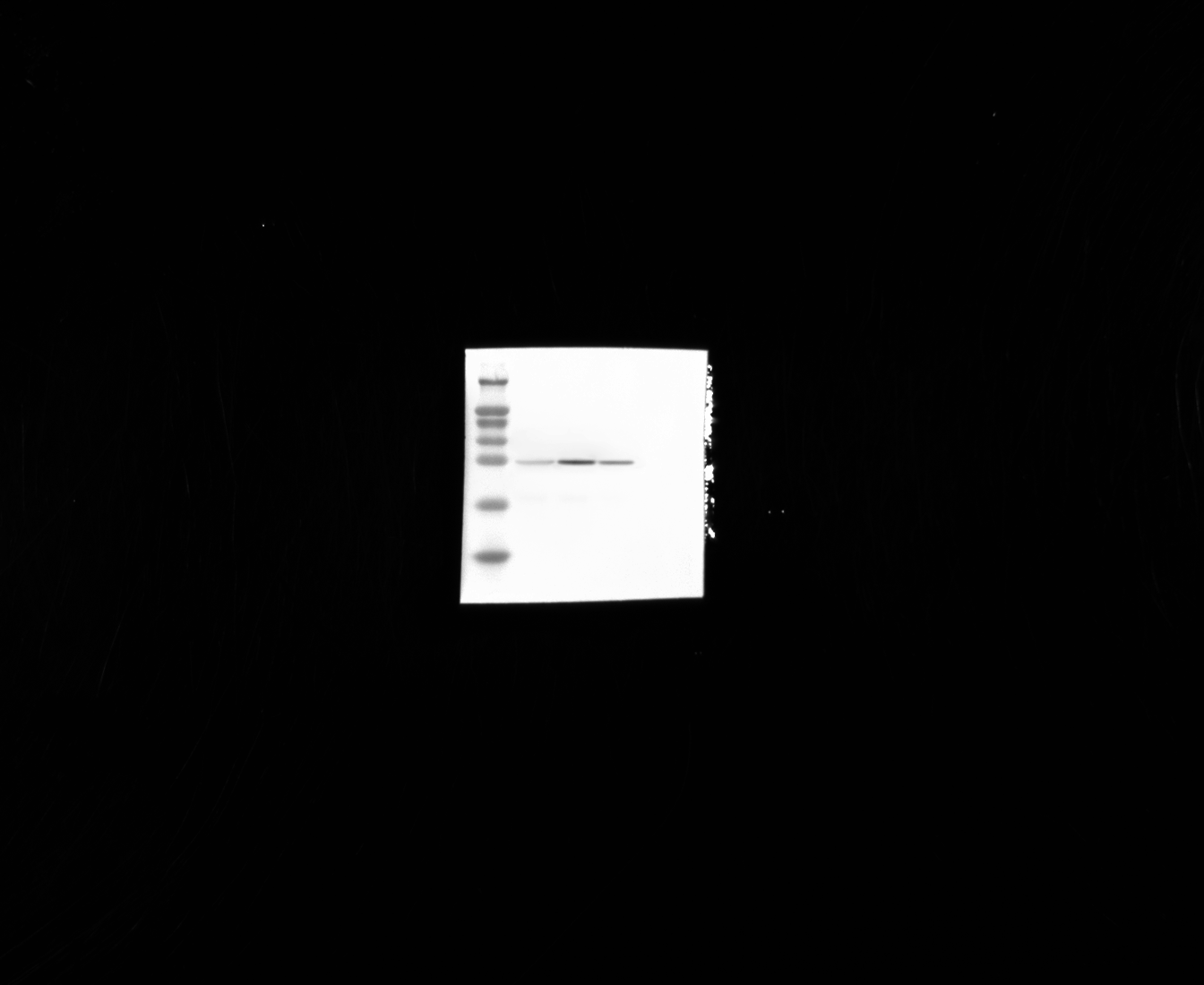


**Il-1B**


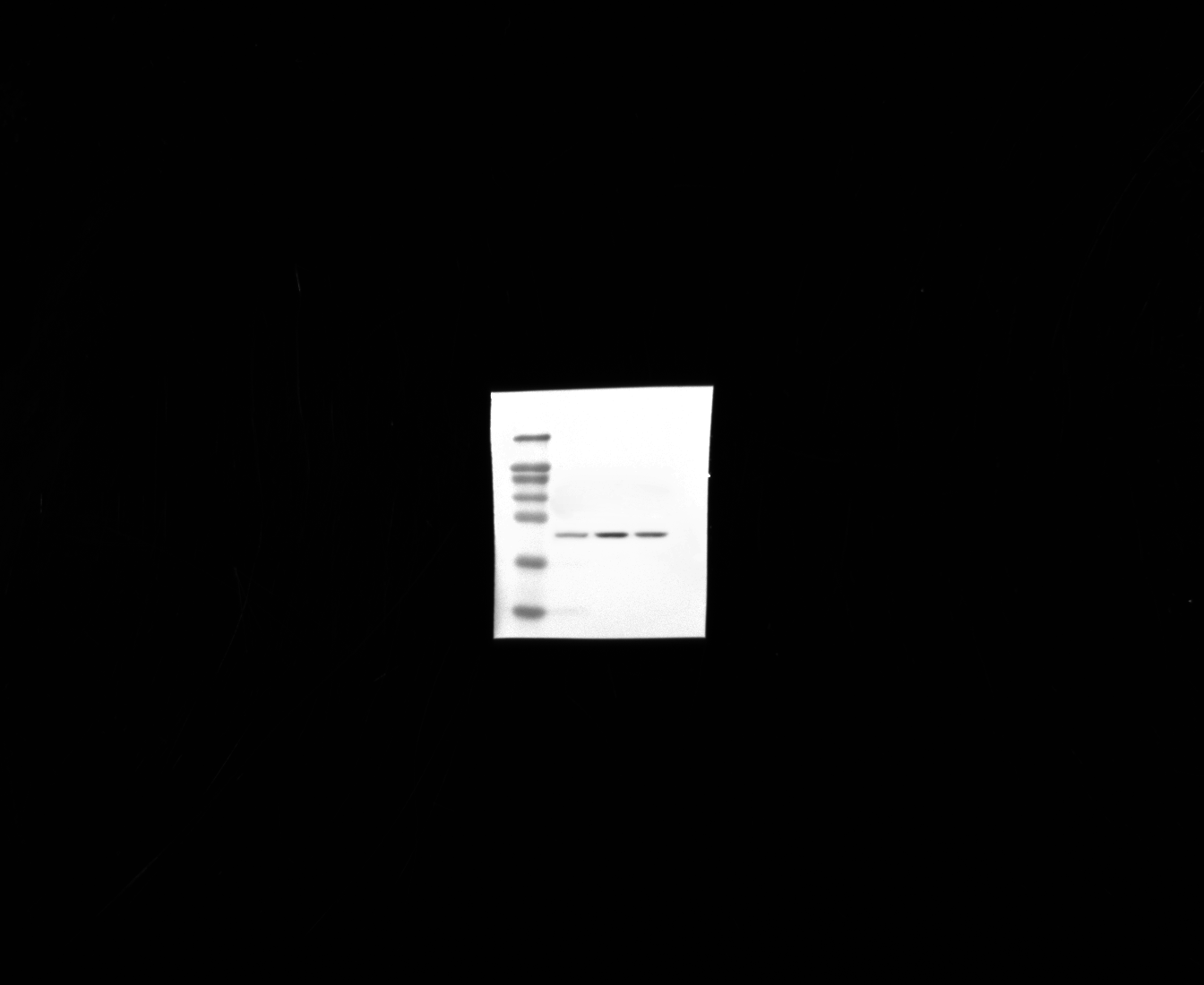


**IL-18**


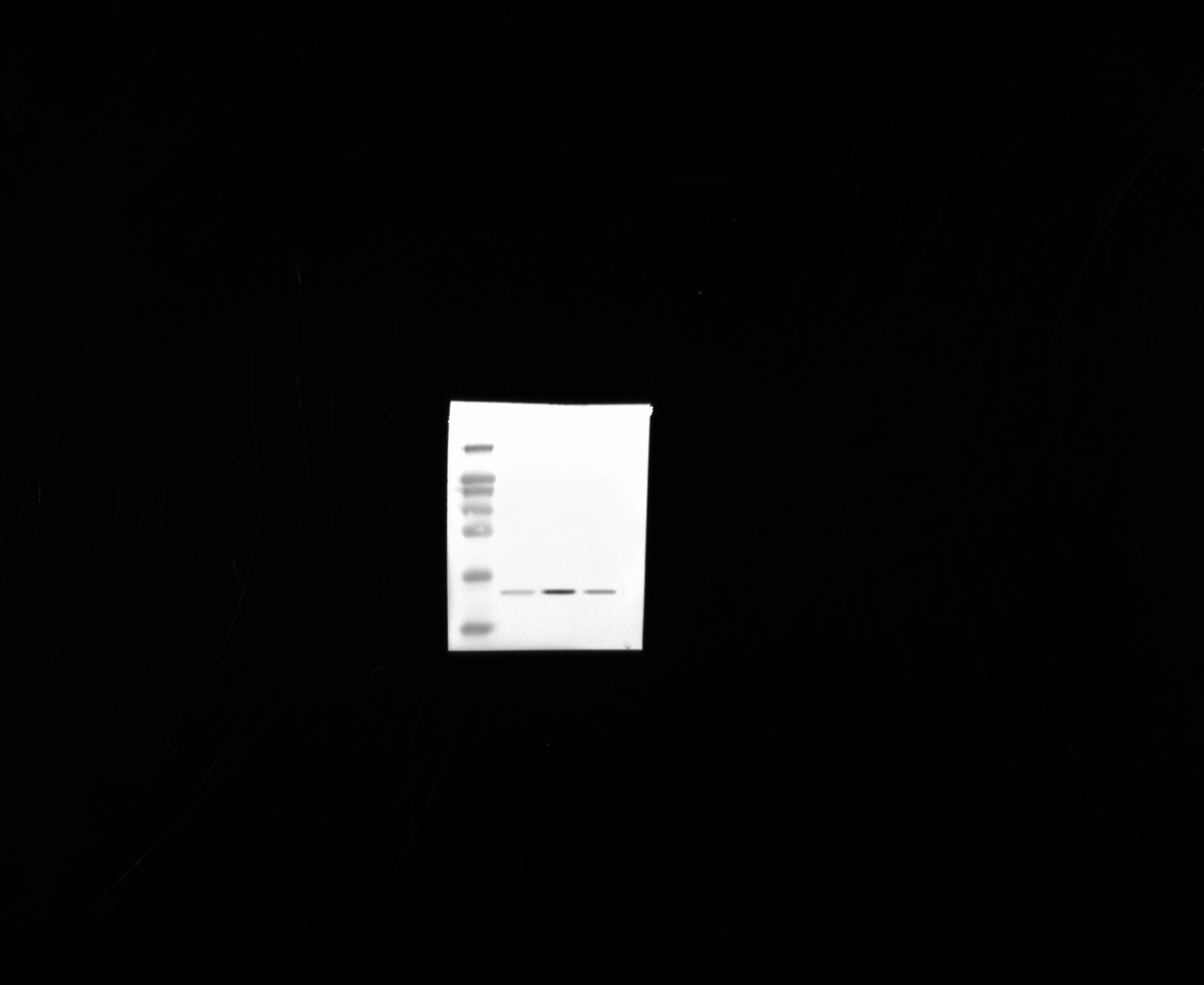


**NLRP3**


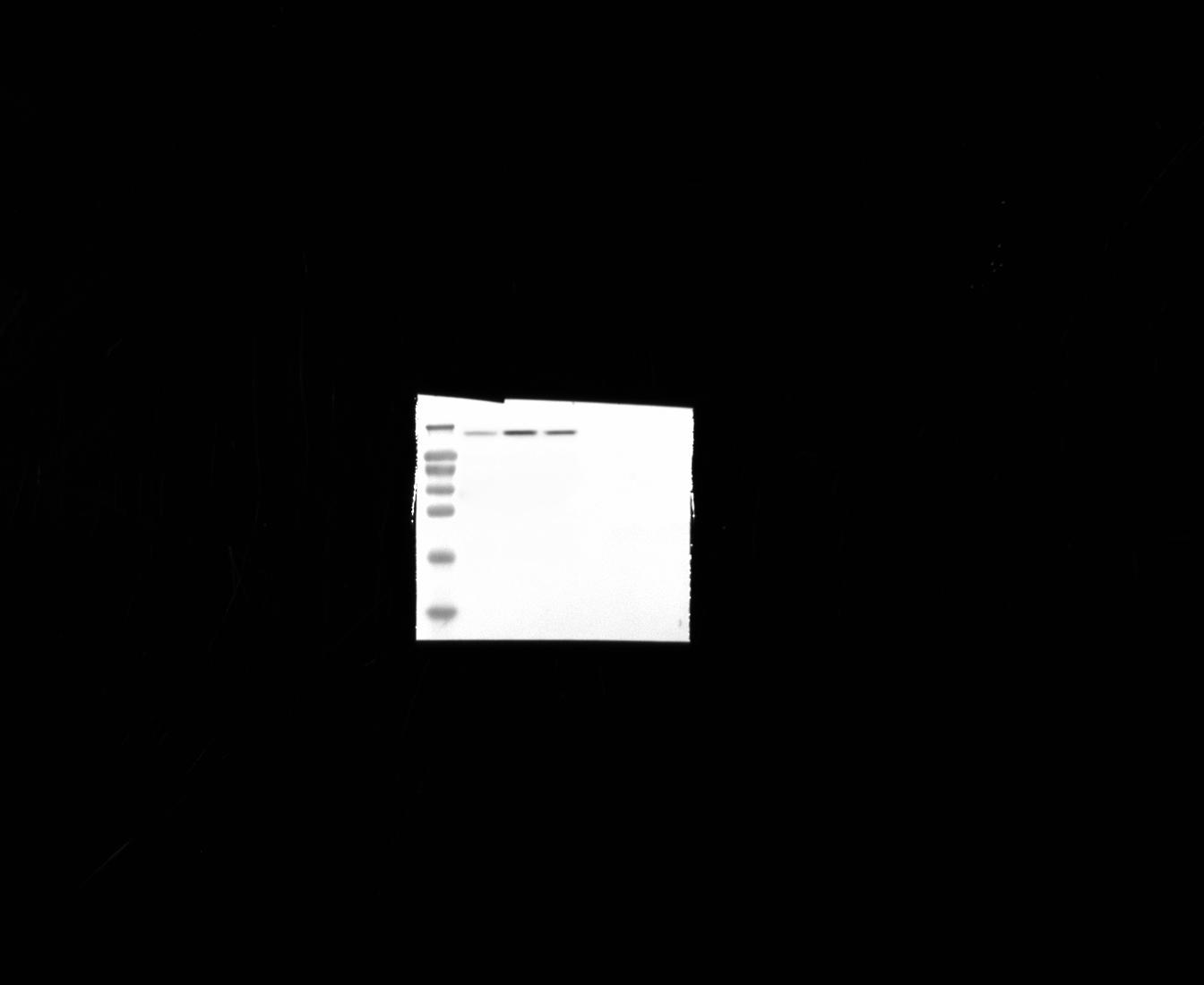

Supplement: Supporting Information — Original western blots associated with this study can be found as Supporting Information. File S1. This file contains the original full-length western blot images and ladder corresponding to the cropped figures presented in the manuscript. [file 7119597.f1.docx]
